# The Aging Trend of Insureds and Stochastic Evaluation of Financial Sustainability of Basic Pension in China

**DOI:** 10.3389/fpubh.2022.911535

**Published:** 2022-05-19

**Authors:** Xiaohua Chen

**Affiliations:** School of Finance, Jiangxi University of Finance and Economics, Nanchang, China

**Keywords:** aging population, basic pension, financial situation, evaluation, stochastic simulation

## Abstract

The document, *National Medium and Long Term Plan for Actively Responding to the Aging of Population* points out that in order to actively respond to the aging population in China, it is necessary to steadily increase the endowment wealth reserves. To achieve this goal, it is urgent to stochastically assess the future financial situation of the basic pension insurance in China, grasp its various possible conditions, trends, and corresponding confidence intervals, so that the government can take targeted measures to gradually consolidate the wealth reserves for this basic insurance first, and then steadily increase the social wealth reserves for the elderly. Thus, this paper first analyzes the characteristics of the aging population of insureds participating in basic pension insurance, and then randomly simulates the long-term financial situation of the basic pension insurance. The study found that the aging of the insureds has the characteristics of “fierce coming and slow decline” and “long-term seriousness.” Among the six indicators of the financial situation of basic pension insurance, Indicator 1 (the current year's expenditures as a proportion of current year's contributions), Indicator 2 (current year's balance of contributions and expenditures), Indicator 3 (current year's payment gap as a proportion of current year's contributions), Indicator 4 (accumulated balance), Indicator 5 (fund ratio), and Indicator 6 (accumulated payment gap as a proportion of current year's contributions) are respectively in the range of [0.73%, 1.80%], [−12.05, −0.12] trillion yuan, [0.29%, 3.89%], [−133.39, −5.62] trillion yuan, [2032, 2043] years and [6.72%, 215.63%] with a probability of 95%. We analyzed the influence direction and degree of main parameters on the financial situation of the fund and analyzed the impact of parameter value paths on the final financial status of the fund to improve the ability to strengthen fund reserve. The backtracking found that if the value path of the average salary growth rate shows a trend of rising first and then falling, then the final financial situation at the end of the period will be “worse.” If it shows a trend of falling first and then rising, the final financial situation will be “better.”

## Introduction

The response to population aging has become a global issue, which not only affects the health and care of the elderly population (1, 2) but also brings challenges to the elderly's retirement life, such as insufficient pension and short supply of the elderly service industry. As for China, the Central Committee of the Communist Party and the State Council initiated the document of *National Medium and Long Term Plan for Actively Responding to the Aging of Population* (the “Plan” for short) in November 2019, which indicates that coping with population, aging has become a national strategy in China. The “Plan” points out that it is necessary to steadily increase the endowment wealth reserves in order to consolidate the social wealth reserves in response to population aging. Basic pension insurance, as a system that covers the whole people and ensures the basic life of retirees, and the tamping degree of its wealth reserves are the premises to realize the steady increase of endowment wealth reserves. Thus, it is urgent to use stochastic technology to evaluate the future financial situation of the basic pension insurance system and master its various possible future financial conditions and their corresponding confidence intervals, which are conducive to the comprehensive regulation and control of the government. First, we gradually consolidated the fund reserves of basic pension insurance, and then achieved the goal of a steady increase in endowment wealth reserves.

From a long-term perspective (the next 72 years), under the complex and changeable economic environment, we analyzed the following factors: What kind of change process will the fund reserves of basic pension insurance undergo? What is the corresponding confidence interval? What will the fund reserve be at the end of the prediction period? Is it seriously insufficient or abundant?

In view of the complex and volatile economic environment, stochastic technology needs to be introduced into the financial evaluation of basic pension insurance. Using stochastic technology to describe the uncertainty of the future value of parameters, which is in line with the complex and changeable environment to the greatest extent. In this way, various possibilities for the long-term fund reserves of the basic pension insurance can be depicted. At present, most studies on the evaluation of the financial situation of China's basic pension insurance use actuarial methods, but they rarely consider the random fluctuation of parameters. These studies can be divided into three categories. The first category is to measure the size of implicit debt (3–5). The second category is to explore the contribution and expenditure gap or financial burden of pension insurance (6–8). The third category is to evaluate the long-term financial status and sustainability of pension insurance (9–14). The actuarial methods used in these studies are worth learning from, but they cannot answer the above-mentioned problems since stochastic technology is not introduced.

There are still few studies on the application of stochastic techniques in China's basic pension insurance. The Lee–Carter model was originally used to predict trends in population mortality dynamics (15). Subsequently, Lee and his collaborators continued to explore and stochastically simulated future population changes in the United States, replacing the “high,” “medium,” and “low” scenarios of traditional population forecasting (16). The “OASDI” report of the United States first used stochastic technology to evaluate the long-term financial situation of public pension insurance in 2003. Today's “OASDI” report mainly uses the randomization technology of standardized time series (17). Learning from these experiences, stochastic technology is gradually applied to the research of basic pension insurance in China. Dong et al. (18) calculated the implicit debt of basic pension insurance considering that both interest rate and mortality are random variables. Wang and Mi (19) considered the random fluctuation of bookkeeping interest rate and salary growth rate in each year, and randomly simulated the individual total substitution rate and net substitution rate of the pension insurance. Sun (20) assumed that retirement age, interest rate, and mortality were random variables, which deduced actuarial functions, such as life annuity and retirement annuity, and numerically simulated the influence of relevant factors on actuarial functions. Zheng and Liao (21) assumed that the payment status and retirement time were random variables that established the contribution and expenditure prediction model for the basic pension insurance. The accuracy of the model was verified by the data of Beijing in 2014, but the parameter setting process was complex, which limited the popularization and application of their model. The randomization methods of relevant parameters in these studies are worth learning from, but they do not directly focus on the long-term financial status or fund reserves of basic pension insurance, and thus do not directly answer the above questions.

Although Tian and Zhao (22) considered the random fluctuations of the fertility rate, mortality, and average salary growth rate and predicted the finance of basic pension in the next 75 years, the model construction did not consider the provisions of the decision of *the State Council Decision on Improving the Public Pension System for Enterprise Employees* (State Council Document 38 of 2005), and the model is relatively rough. Chen and Yang (23) assumed that the bookkeeping interest rate and return on investment (ROI) were random variables that simulated the finances of individual accounts in the next 50 years, and found that individual accounts have better self-balancing. However, these studies only focus on the long-term finance of a certain part of the basic pension insurance for urban employees, and do not examine the long-term fund reserves of the basic pension insurance as a whole (that is, including the basic pension, transition pension, and individual account pension at the same time), so they cannot comprehensively answer the above questions. What is more, the existing studies consider at most three random parameters, and lack comprehensive randomization of suitable parameters, so it is impossible to accurately answer the above questions.

In recent years, the Chinese government has successively issued the *Implementation Plan for Transferring Part of State-owned Capital to Enrich Social Security Funds* and established a central adjustment system for basic pension funds for enterprise employees. The transfer of state-owned capital does not affect the current balance of revenue and expenditure of the basic pension funds but can enrich the accumulated balance. Although the central adjustment system can effectively delay the year when the current payment gap appears in some provinces (24) and play the role of “robbing the rich and helping the poor,” its impact on the total amount of basic pension funds in the whole country is neutral (25). Different from the first two, the *Comprehensive Scheme on Reducing Social Insurance Contribution Rates* in 2019 lowered the contribution rate paid by work units for employees and adjusted the calculation caliber of the average salary of urban employees, which will inevitably affect the contributions and pension expenditures of basic pension insurance. Thus, in the context of the implementation of this comprehensive plan, and assuming that the basic pension insurance is in a more severe environment (i.e., without considering the transfer of state-owned capital and possible future delaying retirement policies to isolate their beneficial effects), we take the basic pension insurance for enterprise employees as an example. According to the *State Council Decision on Establishing Unified Public Pension System for Enterprise Employees* (State Council Document 26 of 1997) and the State Council Document 38 of 2005, the contributions and expenditures of the actuarial prediction model of basic pension insurance in the forecast period is established. Considering the random factors suitable for parameters to simulate the uncertain future environment as much as possible, we obtained various possibilities of long-term fund reserves of basic pension insurance funds by random simulation.

There are five differences between this study and the existing literature. First, this study positively and accurately responds to the possible change trend of long-term fund reserves of China's basic pension insurance and the possible situation of fund reserves at the end of the forecast period. Second, the study conducts a more comprehensive randomization of the appropriate parameters in the contributions and expenditures prediction model of the basic pension insurance, specifically considering nine random variables, more than three random variables from the existing literature. Third, when doing sensitivity analysis, the traditional single change value is replaced by continuously changing the parameter value, so that the sensitivity analysis result is more robust and reliable. Fourth, based on the benchmark case, the influence of the fluctuation of each main parameter on the long-term fund reserve status is simulated one by one and sorted. Fifth, the concept of backtracking is introduced to analyze whether the random value path of the main parameters has certain characteristics when the final fund reserve is “worse” or “better.”

## Actuarial Prediction Model Construction and Parameter Random Setting Method

The fund reserve status of the basic pension insurance is measured by six indicators: Indicator 1 (the current year's expenditures as a proportion of current year's contributions), Indicator 2 (current year's balance of contributions and expenditures), Indicator 3 (current year's payment gap as a proportion of current year's contributions), Indicator 4 (accumulated balance), INDICATOR 5 (fund ratio), and Indicator 6 (accumulated payment gap as a proportion of current year's contributions). Indicator 1 refers to the proportion of pension expenditures in the current year to the pension insurance contribution revenues in the current year, which measures the trend of changes in the fund reserves. Indicator 2 is the flow indicator of the fund reserves, specifically referring to the difference between the pension insurance contribution revenues and the pension expenditures in the current year. Indicator 3 measures the severity of the payment gap in the current year, which refers to the proportion of the scale of the pension payment gap in the pension insurance contribution revenues in the current year.

Indicator 4 is the stock indicator, which refers to the accumulated balance of the fund at the end of the current year. Its calculation formula is as follows: if the accumulated balance of the previous year is negative, the accumulated balance is equal to the accumulated balance of the previous year plus the balance of contributions and expenditures of the current year directly. If the accumulated balance of the previous year is positive, the accumulated balance is equal to the accumulated balance of the previous year × (1 + ROI) + balance of contributions and expenditures of the current year. Indicator 5 measures the actual payment ability of the fund reserves and refers to how long the accumulated funds at the end of the previous year can be used to pay the pension of the current year. Indicator 6 refers to the proportion of the accumulated balance deficit formed in the past in the current year's pension insurance contribution revenues, which means how many times the current year's pension insurance contribution revenues are required to fill the gap in the fund reserves.

According to the State Council Document 26 of 1997, the insureds of basic pension insurance can be subdivided into “old people,” “middle people,” and “new people”. The “old people” refer to the insureds who have retired before he implementation of the document; “middle people” refer to the insureds who joined before the implementation of the document and retired after the implementation; “new people” refer to the employees who participate in the basic pension insurance after the implementation of the document. According to the provisions of the State Council Document 26 of 1997 and the State Council Document 38 of 2005, the balance of contributions and expenditures of basic pension insurance in year *t* is equal to the sum of the balance of contributions and expenditures of the social pooling accounts and the individual accounts in year *t*. The contributions and expenditures balance of the social pooling accounts and individual accounts in year *t* are equal to the difference between the pension insurance contribution revenues and pension expenditures of their corresponding accounts. The pension expenditures in social pooling accounts are equal to the basic pension of the “old people” plus the basic pension, the transition pension, and individual accounts pension exceeding the stipulated payment months of the retired “middle people” plus the basic pension and individual accounts pension exceeding the stipulated payment months of the retired “new people.” The expenditures in individual accounts are equal to the individual accounts pension within the stipulated payment months of retired “middle people” and “new people” plus the return amounts of individual accounts balance when the insureds die.

### Model Symbol Settings

Let the age of persons to become firm employees and participate in the pension be *e*, the retirement age of insureds be *r*, and the ultimate age of insureds be ω. The contribution rate of basic pension insurance paid by enterprises for employees (i.e., enterprise contribution rate) is *b*_*t*_, and the contribution rate paid by employees for themselves (i.e., individual contribution rate) is *c*_*t*_. *L*_*t, x*_ refers to the number of insureds aged *x* in year *t*. ${\bar{S}}_{t}$ is the weighted average salary of urban employees in year *t*. *S*_*t, x*_ is the salary of employees aged *x* in year *t*, and *s* is the growth rate of seniority salary. *B*_*t, x*_, *T*_*t, x*_, and *I*_*t, x*_ are the basic pension, transition pension, and individual accounts pension, respectively, received by retirees aged *x* in *t*. The *i*_*t*_, *j*_*t*_, *g*_*t*_, and ρ_*t*_ are the ROIs, bookkeeping interest rate, average salary growth rate, and pension growth rate in year *t*, respectively. The transitional coefficient to pay transitional benefits is ε. The *z* is the implementation year of the State Council Document 26 of 1997, that is, *z* = 1997. It is assumed that pension contributions and pension expenditures occur at the beginning of each year.

### Contribution Revenues of Basic Pension Insurance Fund

The contribution revenues from social pooling accounts and individual accounts are equal to the pension insurance premiums paid by the employers for the insured employees and paid by the insured employees for themselves, respectively. Since some salary items are included in the statistical salary and not included in the contributory salary, the contributory salary is lower than the statistical salary. The contributory salary is the product of the insured employee's salary in the previous year and the ratio, *d*_*t*_ of the contribution salary to the statistical salary. The contribution revenues from social pooling accounts and individual accounts are as follows:


(1) $$\begin{array}{l} b_{t}\overset{r-1}{\underset{x=e}{\sum }}L_{t,x}\cdot d_{t}S_{t-1,x-1} \end{array}$$

(2) $$\begin{array}{l} c_{t}\overset{r-1}{\underset{x=e}{\sum }}L_{t,x}\cdot d_{t}S_{t-1,x-1} \end{array}$$

The *S*_*t, x*_ can be calculated by the following formula. The *t*_*o*_ is the starting year of the measurement. From $S_{t,x}=\left(1+s\right)S_{t,x-1}=\cdot \cdot \cdot ={\left(1+s\right)}^{x-e}S_{t,e}$, we know ${\bar{S}}_{t}=\overset{r-1}{\underset{x=e}{\sum }}L_{t,x}S_{t,x}/\overset{r-1}{\underset{x=e}{\sum }}L_{t,x}$$=S_{t,e}\cdot \overset{r-1}{\underset{x=e}{\sum }}L_{t,x}\cdot {\left(1+s\right)}^{x-e}/\overset{r-1}{\underset{x=e}{\sum }}L_{t,x}$, then $S_{t,e}={\bar{S}}_{t}\cdot \overset{r-1}{\underset{x=e}{\sum }}L_{t,x}/\overset{r-1}{\underset{x=e}{\sum }}L_{t,x}\cdot {\left(1+s\right)}^{x-e}$ can be deduced. When *t* > *t*_*o*_, ${\bar{S}}_{t}=\overset{t}{\underset{k=t_{o+1}}{\prod }}\left(1+g_{k}\right)\cdot {\bar{S}}_{t_{o}}$; When *t* < *t*_*o*_, we can check the data published in the relevant statistical yearbook to get it. Thus, the *S*_*t, x*_ of employees of each age in year *t* can be obtained, and then the contribution revenues from social pooling accounts and individual accounts can be calculated.

### Benefit Expenditures of Basic Pension Insurance Fund

#### Pension Expenditures of the “Old People”

The age range of “old people” is [*r*+*t–z*, ω]. According to the original regulations, the government only pays the basic pension for the “old people.” The pension of the “old people” aged *x* in *t* is about the salary *S*_*t*−(*x*−*r*)−1, *r*−1_ of the previous year when they retire, multiplied by the pension replacement rate, ${\hat{R}}_{t-\left(x-r\right)}^{\;}$ in the year of retirement. The pension of “old people” is paid by the social pooling accounts, and its expenditures are as follows:


(3) $$\begin{array}{r} PC_{t}^{\;O}=\overset{\omega }{\underset{x=r+t-z}{\sum }}L_{t,x}^{\;}\cdot {\hat{R}}_{t-\left(x-r\right)}^{\;}S_{t-\left(x-r\right)-1,\;r-1}\cdot \\ \overset{t}{\underset{h=t-\left(x-r\right)}{\prod }}\left(1+\rho_{h}\right)/\left(1+\rho_{t}\right) \end{array}$$

#### Pension Expenditures of the “Middle People”

The age range of retired “middle people” is initially [*r, r*+*t–z–*1] years old. When retired “new people” appear, that is, *t* ≥ *z*+2+*l*_*m*_, it becomes [*r*+*t–z–*1–*l*_*m*_, *r*+*t–z–*1] years old, where *l*_*m*_=*r–e–*1, so the age range can be abbreviated as [max(*r, r*+*t–z–*1–*l*_*m*_), *r*+*t–z–*1] years old. The pension expenditures of the retired “middle people” include the expenditures of basic pension, transition pension, and individual accounts pension:


(4) $$\begin{array}{l} PC_{t}^{M}=\overset{r+t-z-1}{\underset{x=\max\left(r,\;r+t-z-1-l_{m}\right)}{\sum }}L_{t,x}^{\;}\cdot \left(B_{t,x}^{\;}+T_{t,x}^{\;}+I_{t,x}^{\;}\right) \end{array}$$

The general formulas of $B_{t,x}^{\;}$, $T_{t,x}^{\;}$ and $I_{t,x}^{\;}$ are shown below, respectively.


(5) $$\begin{array}{l} B_{t,x}^{\;}=\frac{{\bar{S}}_{t-\left(x-r\right)-1}}{2}[1+\frac{1}{\min\left[t-\left(x-r\right)-z,r-e\right]}\underset{\sum_{k=1}\;}{\min[t-\left(x-r\right)-z,\;r-e]}\;\\ \;\frac{d_{t-\left(x-r\right)-k}S_{t-\left(x-r\right)-k\text{-}1,r-k\text{-}1}}{{\bar{S}}_{t-\left(x-r\right)-k-1}}]\\ \;\times \min\left[t-\left(x-r\right)-z,r-e\right]\%\times \frac{\prod_{h=t-(x-r)}^{t}\left(1+\rho_{h}\right)}{1+\rho_{t}} \end{array}$$

(6) $$\begin{array}{l} T_{t,x}^{\;}=\frac{{\bar{S}}_{t-\left(x-r\right)-1}}{t-\left(x-r\right)-z}\\ \;\left(\sum_{k=1}^{t-\left(x-r\right)-z}\frac{d_{t-\left(x-r\right)-k}S_{t-\left(x-r\right)-k-1,r-k-1}}{{\bar{S}}_{t-\left(x-r\right)-k-1}}\right)\cdot \\ \;\left[r-e-\left(t-\left(x-r\right)-z\right)\right]\;\cdot \;\varepsilon \;\cdot \;\frac{\prod_{h=t-(x-r)}^{t}\left(1+\rho_{h}\right)}{1+\rho_{t}} \end{array}$$

(7) $$\begin{array}{l} I_{t,x}^{\;}=I_{t-\left(x-r\right),r}^{\;}=\frac{12}{m_{r}}\times \overset{t-\left(x-r\right)-1}{\underset{k=\max\left(z,\;t-\left(x-r\right)-1-l_{m}\right)}{\sum }}\\ \;\left[c_{k}d_{k}S_{k-1,x+\left(k-1\right)-t}\cdot \overset{t-\left(x-r\right)-1}{\underset{h=k}{\prod }}\left(1+j_{h}\right)\right] \end{array}$$

Where the stipulated payment months for individual accounts pension is *m*_*r*_. The individual accounts pay of the individual accounts pension within the stipulated payment months of retired “middle people” is calculated using the formula $PC_{t}^{\;M}$. The social pooling accounts pay basic pension, the transition pension, and individual accounts pension exceeding the stipulated payment months of the retired “middle people” are calculated using the formula $PC_{t}^{\;M}$.

#### Pension Expenditures of the “New People”

The age range of retired “new people” is [*r, r*+*t–z–*l–*l*_*m*_*-*1] years old, that is [*r, t–z*+*e–*1] years old. The “new people” continue to replace “old people” and “middle people” as the years go by. When *t* ≥ *z–e*+ω +1, all the retired people are “new people”, and the “old people” and retired “middle people” are disappeared successively. The pension expenditures, $PC_{t}^{\;N}$of the retired “new people” include basic pension and individual accounts pension.


(8) $$\begin{array}{l} PC_{t}^{N}=\overset{t-z+e-1}{\underset{x=r}{\sum }}L_{t,x}^{\;}\cdot \left(B_{t,x}^{\;}+I_{t,x}^{\;}\right) \end{array}$$

Where $B_{t,x}^{\;}$ and $I_{t,x}^{\;}$ are consistent with the corresponding expressions of formula (5) and formula (7), respectively. The social pooling accounts pay basic pension and individual accounts pension exceeding the stipulated payment months of the retired “new people” are calculated using the formula $PC_{t}^{\;N}$. The individual accounts pay of the individual accounts pension within the stipulated payment months of the retired “new people” are calculated using the formula, $PC_{t}^{\;N}$.

#### Pension Expenditures of Individual Accounts Balance Refund

The individual accounts balance refund $I_{t}^{D}$ include the pension expenditures of the insured in-service workers (i.e., in-service “middle people” and “new people”) when they die, and retirees (i.e., retired “middle people” and “new people”) when they die within the stipulated payment months.


(9) $$\begin{array}{l} \begin{array}{c} I_{t}^{D}=\overset{r\text{-}1}{\underset{x=e}{\sum }}\overset{\min\left(t-z,x-e\right)}{\underset{k=0}{\sum }}\left[c_{t-k}d_{t-k}S_{t-k-1,x-k-1}\cdot \frac{\overset{k}{\underset{n=0}{\prod }}\left(1+j_{t-n}\right)}{\left(1+j_{t}\right)}\right]\cdot D_{t,x}\\ +\overset{\min\left(r+t-z-1,\;r+\left[m_{r}/12\right]-1\right)}{\underset{x=r}{\sum }}\left(\frac{m_{r}}{12}-x+r-1\right)\cdot I_{t,x}\cdot D_{t,x} \end{array} \end{array}$$

Where *D*_*t, x*_ is the number of insureds who died at the age of *x* in year *t*.

### Random Setting Method of Main Parameters

The purpose of parameter randomization is to obtain the values of parameters and their corresponding random fluctuations in each year (*t* > *t*_0_) of the forecast period through standardized time series techniques or other applicable randomization methods based on historical data. The “OASDI” report of the United States uses standardized time series technology (ARIMA) to randomize parameters, such as fertility rate, real salary growth rate, and the number of legal immigrants and immigrants. Wang and Mi (19) used lognormal distribution to fit random distributions of average salary growth rate and bookkeeping interest rate. Xie and Wu (26) and Zhao et al. (27) proved that the Vasicek model can better fit the volatility behavior of China's interest rate market. Then Chen and Yang (23) used Vasicek model to fit the ROI of pension funds, so different parameters have different randomization methods.

Drawing from the experience of previous studies (17, 19, 23), and according to the actual situation of China's basic pension insurance, the values of parameters, such as the growth rate of seniority salary and the transition coefficient are relatively fixed, which are not suitable for randomization. Thus, the following nine parameters are selected as the main parameters and randomized, which are mortality, urbanization rate, unemployment rate, average salary growth rate, ROI, bookkeeping interest rate, pension growth rate, enterprise contribution rate, and individual contribution rate. The suitable randomization methods for each parameter are different. Mortality was randomized using the Lee–Carter model (15, 16). The urbanization rate, unemployment rate, average salary growth rate, and pension growth rate were randomized using standardized time series techniques (17). The ROI was randomized using the Vasicek model (23, 27). The bookkeeping interest rate is assumed to be lognormally distributed (19). The enterprise contribution rate and individual contribution rate of basic pension insurance are assumed to be normally distributed.

## Basic Assumptions and Dynamic Estimation of Parameters

### Basic Assumptions

According to *China population and Employment Statistical Yearbook 2020*, the age composition of urban employees in the first group (16–19 years old) accounts for only 0.8%, and that in the second group (20–24 years old) accounts for 6.8%. With the improvement of the education level of employees, the age *e* of employees who get the first job can be set as the starting age of the second group, which is 20 years old. The current legal retirement age, *r* of male workers, female workers, and female cadres are 60, 50, and 55, respectively. According to the provisions of the State Council Document 38 of 2005, the corresponding individual accounts pension payment months, *mr* are 139, 195, and 170 months, respectively. The maximum statistical age of the national population by age and gender in the *China Population and Employment Statistical Yearbook* over the years is 100 years old, so the ultimate age ω of the insureds is set to 100 years old.

The growth rate of seniority salary *s* is set to be 1.363% (8). The pension transition coefficient ε is generally controlled between 1 and 1.4% (28), and we take the middle value of 1.2%. The annual report on *China's social insurance development 2015* announced that the official pension replacement rate for enterprise retirees is about 67.50%. According to the *China Human Resources and Social Security Yearbook 2018*, the average retirement benefits of employees in 2017 and the average salary of on-the-job employees in the previous year were 29,880 yuan and 72,703 yuan, respectively. We divided the average retirement benefits by the current official pension replacement rate of 67.5%, and then divided it by the average salary of on-the-job employees, the proportion *d*_*t*_ of contribution salary in statistical salary is 60.89%, which is assumed to remain unchanged during the forecast period. Since the earliest year of the data on the average retirement benefits of employees in *China Human Resources and Social Security Yearbook 2018* is 1998, the data of that year are used to estimate the replacement rate,$\hat{R}$of “old people” pension. From the yearbook,the average retirement benefits of retirees in 1998 and the average salary of on-the-job workers in the previous year were found to be 5,304 yuan and 7,405 yuan, respectively. By dividing the average retirement benefits by the average salary of on-the-job employees in the previous year, $\hat{R}$ is calculated as 71.63%, and it is assumed that the pension replacement rate of “old people” is the same.

### Parameter Dynamic Estimation

#### Estimated Number of Insureds

The number of in-service insureds aged *x* in *t* years is equal to the product of the corresponding age population, *P*_*t, x*_ in the national population distribution by age and sex in that year and the labor participation rate, urbanization rate, pension insurance coverage rate, and the proportion of the number of on-the-job enterprises' employees in the number of on-the-job employees in cities and towns of China. In the same way, the number of retired insureds aged *x* in year *t* can be obtained. Then, we can estimate the number of insured persons as *L*_*t, x*_ in each year. The mortality *q*_*t, x*_ (the mortality of *x*-year-olds in year *t*) is multiplied by *L*_*t, x*_ to obtain the number of insureds *D*_*t*_,_*x*_ who died by age and sex in each year.

Estimates of the national population distribution by age and sex *(P*_*t, x*_*)*. According to the data of the United Nations Population Division in 2019, China's net migrant population in 2018 was −1,742,000, accounting for only 0.125% of the total population, with little impact. Thus, the international migration will not be considered for the time being. The cohort element method is used to build a population forecasting model. Without considering the immigration and emigration, the general form of this model is as follows:


(10) $$\begin{array}{l} \{\begin{array}{l} P_{t,0}=\frac{SRB_{t}}{1+SRB_{t}}\cdot \overset{49}{\underset{x=15}{\sum }}P_{t,x}^{F}\cdot FR_{t,x}+\left(1-\frac{SRB_{t}}{1+SRB_{t}}\right)\cdot \overset{49}{\underset{x=15}{\sum }}P_{t,x}^{F}\\ \;\cdot FR_{t,x},\;x=0\\ P_{t,x}=P_{t-1,x-1}\cdot \left(1-q_{t,x}\right),\;x>0 \end{array} \end{array}$$

Where $P_{t,x}^{F}$ is the number of women of childbearing age at the age of *x* in year *t*, The *FR*_*t, x*_ is the fertility rate of women of childbearing age at the age of *x* in year *t*, and *SRB*_*t*_ is the sex ratio of newborns in year *t*.

From the *China Population and Employment Statistical Yearbook 2020*, we know about the national population distribution by age and gender in 2019. The population distribution by age is divided by the corresponding sampling ratio, and the result is used as the initial population distribution. The *FR*_*t, x*_ of each year in the forecast period is calculated from $TFR_{t}\cdot {\bar{h}}_{t,x}$, where *TFR*_*t*_ is the total fertility rate in year *t*, and *h*_*t, x*_ is the standardized fertility coefficient. The total fertility rate, *TFR*_*t*_ for each year of the forecast period refers to the results of the intermediate scenario forecast by the United Nations Population Division in 2019.

The average fertility rate, *FR*_*t, x*_ of urban women of childbearing age by age from 2000 to 2019 is obtained from the *China Population and Employment Statistical Yearbook*. The total fertility rate of the corresponding year is obtained by the formula, $TFR_{t}=\overset{49}{\underset{x=15}{\sum }}FR_{t,x}$, and then the standardized fertility coefficient of these 20 years are obtained by the formula, _*h*_*t, x*_ = *FR*_*t, x*_/*TFRt*_, taking their mean value as the standardized fertility coefficient, ${\bar{h}}_{t,x}$ of each year in the prediction period. The sex ratio, *SRB*_*t*_ of newborns in each year of the prediction period also refers to the intermediate program results predicted by the United Nations Population Division in 2019.

Estimation of dynamic mortality *q*_*t, x*_. The general form of the Lee—Carter model is ln (*m*_*x, t*_) = α_*x*_+β_*x*_*k*_*t*_ +ε_*x, t*_, where *m*_*x, t*_ represents the central mortality of the *x*-year-old at *t*, α_*x*_ refers to the average mortality of the *x*-year-old, β_*x*_ refers to the sensitivity of age *x* to changes in mortality, *k*_*t*_ refers to the degree of change in mortality over time, ε_*x, t*_ is the error term, and it obeys *N*(0, $\sigma_{\varepsilon }^{2}$) distribution. The weighted least squares method (29) was used to fit the parameters, α_*x*_ and β_*x*_, and the double stochastic process (30) was used to fit and predict the parameter, *k*_*t*_. The sample data of national age-specific sex mortality from 1994 to 2019 used to fit the above parameters are derived from the data of *China population and Employment Statistical Yearbook* and the fifth and sixth national censuses over the years. For the absence of the mortality of the elderly in the statistical yearbook, the Coale–Kisker method was used to estimate (31).

Dynamic estimates of the unemployment rate, *u*_*t*_ and the urbanization rate, *ub*_*t*_ in each year of the forecast period. The historical data on the unemployment rate from 1978 to 2019 are obtained from the *China Statistical Yearbook of Population and Employment 2020*, and the historical data on the urbanization rate from 1980 to 2019 are obtained from the *China Statistical Yearbook 2020*. The ARIMA model is used to fit the historical data of the unemployment rate, *u*_*t*_ and the urbanization rate, *ub*_*t*_ respectively, and the obtained model estimation formulas are:


(11) $$\begin{array}{l} \Delta u_{t}=0.5851\Delta u_{t-1}+\varepsilon_{t},\varepsilon_{t}\sim N\left(0,0.2616^{2}\right) \end{array}$$

(12) $$\begin{array}{l} \Delta ub_{t}\text{​}=\text{​}1.0476+0.7042\Delta \;ub_{t-1}\text{​}+\text{​}\varepsilon_{t},\text{​}\varepsilon_{t}\sim N(0,0.2815^{2})\; \end{array}$$

**Figure 1:**
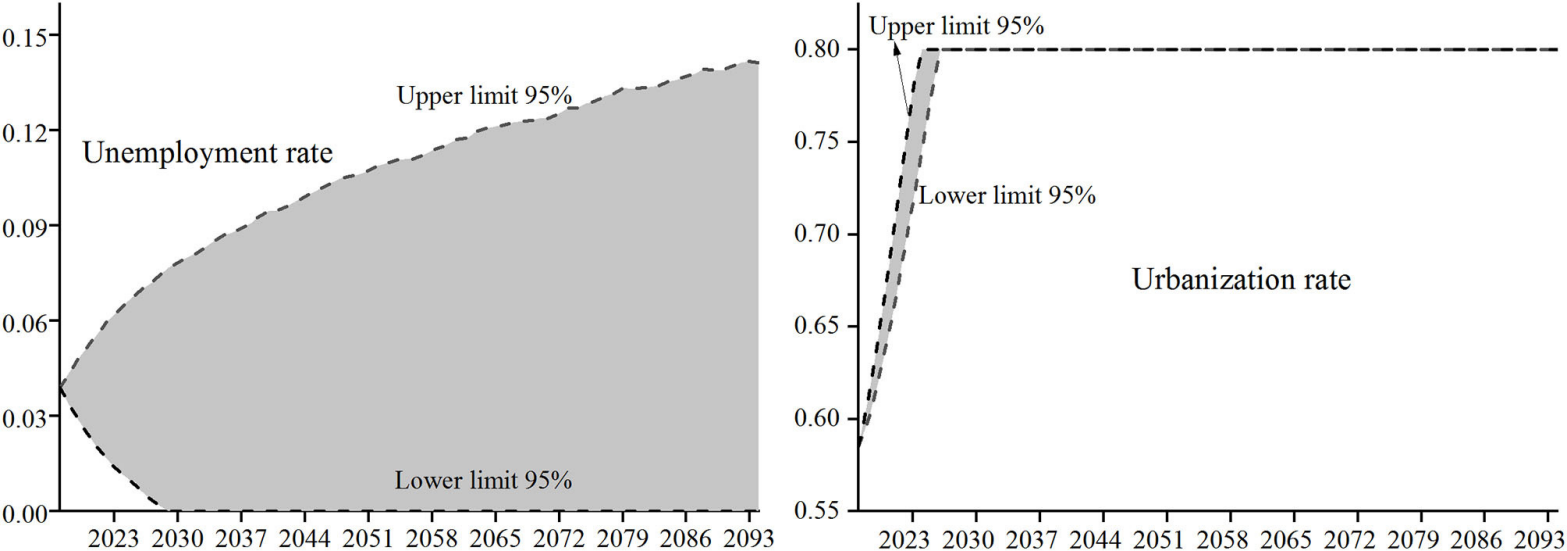
Simulated distribution of unemployment rate and urbanization rate.

Since the lower limit of the unemployment rate is 0, the fluctuating unemployment rate obtained by formula (11) is ≥ 0. After obtaining the fluctuation of the unemployment rate in each year of the forecast period, since the labor participation rate = 1 – unemployment rate, the fluctuation of the labor participation rate are also obtained. Wang and Ge (32) believed that the upper limit of China's urbanization rate is 80%, so the upper limit is taken when the fluctuation range of urbanization rate obtained from formula (12) exceeds 0.8. The 5,000 times Monte Carlo simulation distribution of unemployment rate and urbanization rate in each year of the prediction period are shown in Figure 1.

Estimation of other parameters. Referring to previous practical experience, the pension insurance coverage rate will increase by one percentage point year by year from 85% in 2015 to 95% in 2025 and will remain unchanged in subsequent years; the number of female workers is four times that of female cadres. From the *China Human Resources and Social Security Yearbook* 2020, we come to know that the number of insured employees in enterprises over the years accounted for the proportion of insured employees in urban areas across the country, and the average value of 93.70% is taken as the value of this ratio in each year of the forecast period. In the same way, the average ratio of the number of the retired insureds in the enterprise to that of the national urban retired insureds is 94.40%, which is the value of each year in the forecast period.

#### Estimation of Average Salary Growth Rate and Pension Growth Rate

According to the *China Human Resources and Social Security Yearbook 2020*, we come to know about the average salary of on-the-job employees of enterprises from 1997 to 2019, and then we get the historical data of the average salary growth rate, *g*_*t*_ from 1998 to 2019. Fitting them with ARIMA model, the obtained estimation formula is as follows:


(13) $$\begin{array}{l} \Delta g_{t}=-0.0010-0.3095\Delta g_{t-1}+\varepsilon_{t},\varepsilon_{t}\sim N\left(0,0.0258^{2}\right) \end{array}$$

Due to the rigidity of salary, that is, the decline of salary is sticky, the lower limit of the fluctuation of the average salary growth rate obtained from formula (13) is not <0. In addition, the report of the 19th CPC National Congress in 2017 pointed out that China's economy has turned to a high-quality development stage, and the economic growth has been gradually slowing down. Since the average salary growth rate is closely related to the economic growth rate, it is assumed that the average salary growth rate in the forecast period is not > the average salary growth rate of 9.20% in 2017. Thus, the average salary growth rate in the forecast period fluctuates between 0% and 9.20%.

According to *China Demographic and Employment Statistical Yearbook 2020*, the average salary and number of urban nonprivate sector employees and the average salary and number of urban private sector employees at the end of 2019 can be obtained, respectively. It can be estimated that the weighted average salary ${\bar{S}}_{t}$ of urban unit employees in the 2019 is 63,182 yuan, which will increase by *g*_*t*_ in years after 2019. The ${\bar{S}}_{t}$ in the years before 2019 is the average salary of the on-the-job employees of the enterprise in the corresponding year published in the statistical yearbook, so ${\bar{S}}_{t}$ can be obtained. The pension growth rate, ρ_*t*_ is generally 60–80% of the average salary growth rate, and 80% is taken here (25), so the fluctuation distribution of the pension growth rate can also be obtained.

#### Estimation of ROI

The Vasicek model is used for fitting, and a relatively active and representative 30-day “interbank pledged repo weighted interest rate” is used as a sample of ROI *i*_*t*_ (23). The data are obtained from the observed values of trading days from January 4, 2015 to September 30, 2021. The estimated Vasicek model formula is:


(14) $$\begin{array}{l} i_{t}=0.0015002+0.95275i_{t-1}+0.0020282dW_{t} \end{array}$$

Where *W*_*t*_ is the standard Brownian motion, and *dW*_*t*_ (i.e., *W*_*t*_–*W*__*t*−_1_) obeys the standard normal distribution. In September 2021, the National Council of Social Security Funds issued the 2020 annual report on the entrusted operation of the basic pension insurance fund, pointing out that the average annual ROI of the fund since the entrusted investment is 6.89%, which is the initial ROI. Then the ROI of each year in the prediction period can be obtained from formula (14).

#### Estimation of Bookkeeping Interest Rate

It is assumed that the fluctuation of bookkeeping interest rate follows a lognormal distribution (19). Since China's General Office of the Ministry of Human Resources and Social Security and the General Office of the Ministry of Finance began to publish the bookkeeping interest rate of individual accounts of basic pension insurance for urban employees in 2016, the one-year deposit interest rate of the bank over the years in the website of the people's Bank of China can be used as the bookkeeping interest rate for the year 2015 and previous years with reference to practical experience. The maximum likelihood method is used for fitting, and the estimated lognormal distribution result is ln (–3.173, 0.364).

#### Estimation of Contribution Rates

The enterprise contribution rate, *b*_*t*_ of the basic pension insurance is reduced from 20% stipulated in the State Council Document 38 of 2005 to 16% of the *Comprehensive Plan for Reducing Social Insurance Rates* (the document 13 of 2019 issued by the General Office of the State Council). The pace of adjusting enterprise contribution rate in various provinces to achieve this goal may be different, so the enterprise contribution rate, *b*_*t*_ will fluctuate between 20 and 16%. In order to further reduce the payment burden of enterprises, the enterprise contribution rate, *b*_*t*_ may even drop to 12% in the future. Considering the current situation of the enterprise contribution rate *b*_*t*_, the probability of its fluctuation value of 16% should be the maximum, so it can be assumed that it obeys a normal distribution with a mean value of 16% and a fluctuation probability of 95% in the range of 12–20%. The meaning of this distribution is that the enterprise contribution rate fluctuates by 4 percentage points around 16% with a 95% probability. Looking at the standard normal distribution function table, it can be calculated that its standard deviation is (20–16%)/1.96, which is equal to 0.0204, so *b*_*t*_obeys the normal distribution of *N* (0.16, 0.0004).

Similarly, according to the State Council Document 26 of 1997 and the State Council Document 38 of 2005, the individual contribution rate, *c*_*t*_ of basic pension insurance has changed from the previous 11 to 8%. When the individual contribution rate is allowed to fluctuate, it can be assumed that this rate follows the normal distribution with a mean of 8%, and the probability of fluctuating around 3 percentage points of 8% is 95%. It can be seen that the standard deviation of this distribution is 0.0153, so the *c*_*t*_ follows the normal distribution of *N*(0.08, 0.0002).

## Aging Characteristics of Insureds and Financial Status of Basic Pension Fund

### Aging Characteristics of Insureds

**Figure 2:**
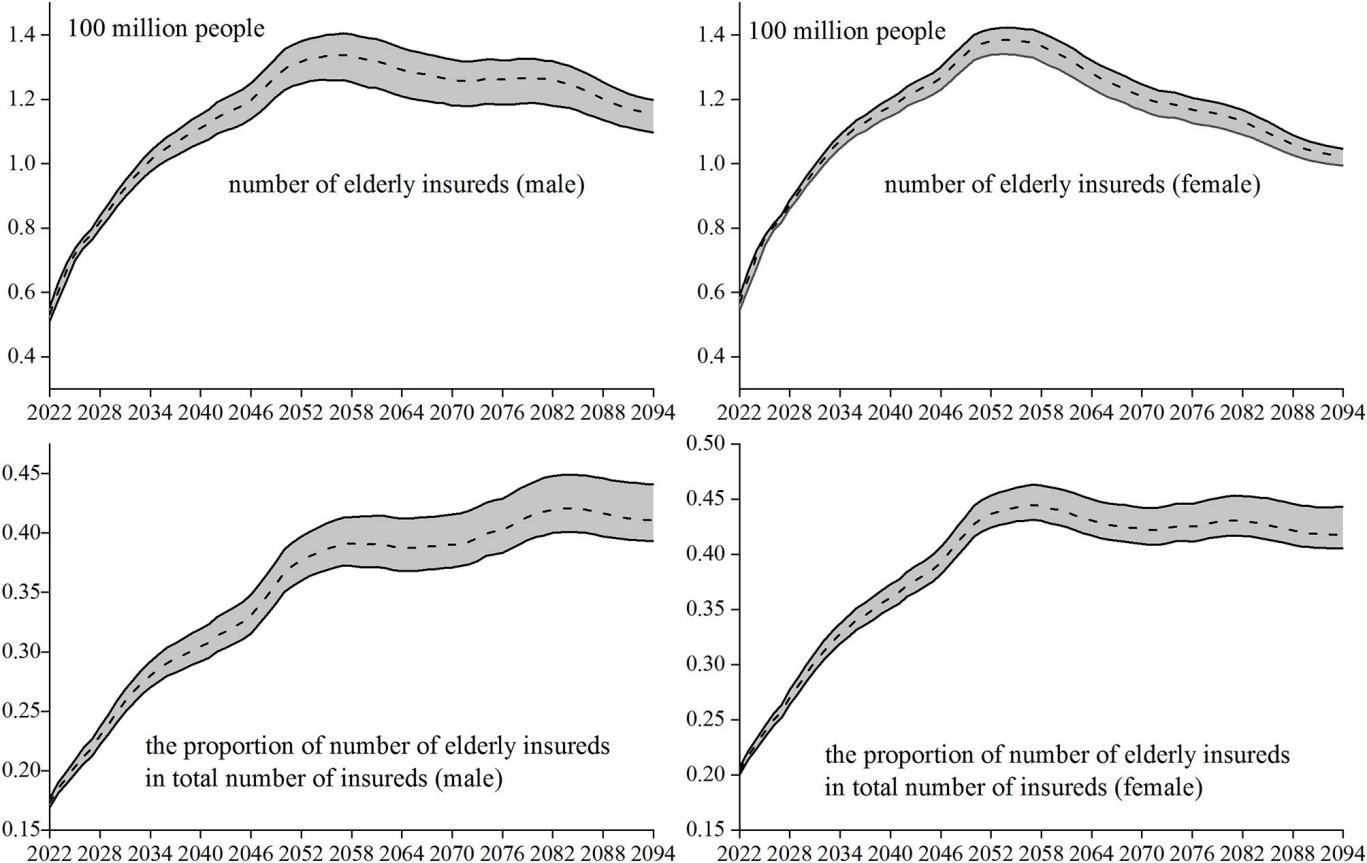
Aging characteristics of insureds.

The insured elderly population refers to the insureds, aged 60 and above. The trend of changes in the number of elderly insureds is used as an indicator to measure the aging characteristics of the insureds. The proportion of population aging generally refers to the proportion of people aged 60 and above in the total number of people. Similarly, the proportion of the number of the elderly insureds in the total number of insureds is taken as another important indicator to measure the aging characteristics of insureds. According to the above estimation of the number of insureds and the parameter setting method, the number of elderly insureds in each year of the prediction period and its proportion in the total number of insureds can be obtained. The variation trend (dotted line in the figure) and its 95% confidence interval (between the two solid lines in the figure) obtained from 5,000 Monte Carlo simulations of the two indicators are shown in Figure 2.

In the early forecast period, the number of elderly insureds (male and female) has been rising rapidly. The number of women will reach the peak of aging around 2,053, while the number of men will be about 4 years later than that of women. In the following years, the number of elderly insureds will slowly decline, and the decline in the number of women is slightly > that of men. It may be that the family planning policy implemented in China in the early stage and the rising cost of living year by year have led to the continuous decline of the fertility rate, resulting in the gradual reduction of the population participating in work and insurance, which further led to the slow downward trend of the elderly insureds retired around 2,050. The number of elderly insureds show the characteristics of a rapid increase at first and then a slow decline, indicating that the aging of the insureds has the characteristics of “fierce coming and slow decline.”

From the perspective of the proportion of the number of elderly insureds among the total number of insureds, in the early stage of the forecast and the proportions of men and women are rising rapidly with a diagonal slope of about 45 degrees. Until 2057, the proportion of different genders shows different trends. This proportion of women fluctuated smoothly, to about 0.42 in 2094; while this proportion of men was still rising, slowing down, and falling back halfway, to about 0.41 by 2094. Although the aging of the insureds has the characteristics of “fierce coming and slow decline,” the decline stage has not significantly reduced the value of this proportion, so the aging of the insureds also has the characteristics of “long-term seriousness,” Then, based on these two characteristics of the elderly insureds, what kind of change trend will the long-term fund reserves of basic pension insurance show?

### Fund Reserves Under the Scenario of Fixed Parameter Values

The research scenario without considering the random fluctuation of parameters is called the fixed parameter value scenario. Taking the mean value of the random distribution of the above nine main parameters, the fixed values of these parameters in each year of the prediction period can be obtained. For example, in formula (11), the formula is Δ *u*_*t*_ = 0.5851 Δ *u*__*t*_−1_ after taking its mean value. Combined with the values of other non-random parameters, the fund reserve status under the fixed scenario can be obtained. According to *China's Labor Statistics Yearbook 2021*, the initial cumulative amount of basic pension insurance fund for enterprise employees in 2020 is 4,440.17 billion yuan. In the absence of continuous financial subsidies, the calculation results of the six indicators (Indicator 1: the current year's expenditures as a proportion of current year's contributions, Indicator 2: current year's balance of contributions and expenditures, Indicator 3: current year's payment gap as a proportion of current year's contributions, Indicator 4: accumulated balance, Indicator 5: fund ratio, and Indicator 6: accumulated payment gap as a proportion of current year's contributions) to measure the status of long-term fund reserves are shown in Table 1.

**Table 1:** Fund reserve status under fixed parameter value scenario.

**Year**	**Indicator 1**	**Indicator 2 (trillion yuan)**	**Indicator 3**	**Indicator 4 (trillion yuan)**	**Indicator 5**	**Indicator 6**
2022	0.86	0.73	—	9.19	1.75	—
2023	0.90	0.58	—	10.07	1.65	—
2024	0.93	0.44	—	10.83	1.55	—
2025	0.96	0.28	—	11.46	1.49	—
2026	0.99	0.10	—	11.93	1.43	—
2027	1.00	−0.04	0.01	12.27	1.34	—
2028	1.03	−0.27	0.03	12.39	1.23	—
2029	1.06	−0.56	0.06	12.23	1.11	—
2034	1.17	−2.19	0.17	6.26	0.38	—
2037	1.21	−3.41	0.21	−2.25	0.00	0.14
2039	1.25	−4.53	0.25	−10.71	0.00	0.60
2044	1.33	−8.03	0.33	−43.68	0.00	1.82
2049	1.46	−14.28	0.46	−100.64	0.00	3.24
2054	1.54	−21.42	0.54	−194.72	0.00	4.87
2059	1.55	−28.32	0.55	−323.20	0.00	6.27
2064	1.54	−35.85	0.54	−486.34	0.00	7.38
2069	1.58	−47.66	0.58	−698.13	0.00	8.54
2074	1.67	−65.57	0.67	−986.02	0.00	10.02
2079	1.75	−87.16	0.75	−1375.59	0.00	11.85
2084	1.80	−108.84	0.80	−1878.29	0.00	13.86
2089	1.81	−127.51	0.81	−2478.29	0.00	15.74
2094	1.85	−152.70	0.85	−3187.41	0.00	17.69

Indicator 1 (the proportion of current year's expenditures to current year's contributions) continued to rise during the forecast period, resulting in the continuous decline of Indicator 2 (current year's balance of contributions and expenditures) and Indicator 4 (accumulated balance). The fund reserves of basic pension insurance will face the dilemma of “income does not cover expenditure” in 2027, that is, there will be a payment gap in the current year. After that year, Indicator 3 (the proportion of current year's payment gap to current year's contributions) will increase year by year, and it will rise to 0.85 in 2094. The accumulated balance or fund ratio (Indicator 5) indicates that its wealth reserves will be exhausted in 2037, and Indicator 6 (the proportion of accumulated payment gap to current year's contributions) in subsequent years rises rapidly, reaching as high as 17.69 in 2094. It can be seen that the fund reserves of basic pension insurance will be seriously insufficient in the long run, and the conclusions are similar to previous studies (14, 22, 33).

### Fund Reserves Under Random Scenario of Parameter Values

**Figure 3:**
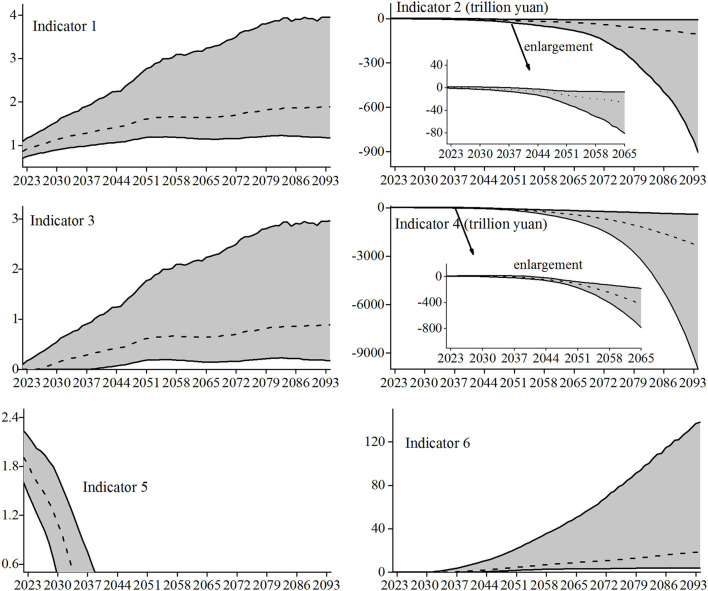
Change trend and confidence interval of long-term fund reserves.

The random fluctuations of nine main parameters, such as mortality, urbanization rate, unemployment rate, average salary growth rate, etc. are considered to simulate the various possibilities of long-term fund reserves of basic pension insurance under complex and changeable environment to the greatest extent. They are recorded as the random scenario of the parameter value. Based on MATLAB software and Monte Carlo technology, 5,000 random simulation results of the long-term fund reserve status of the basic pension insurance are obtained. The change trend and 95% confidence interval of the six indicators during the prediction period are shown in Figure 3.

The change trend (dotted line in the figure) of the six indicators measuring the long-term fund reserves under the scenario of random-parameter value is basically consistent with the change trend of each indicator under the scenario of fixed-parameter value, but the random scenario of the parameter value can provide very rich information on the change trend of fund reserves. For example, the average annual growth rate to measure the trend of Indicator 1 (the current year's expenditures as a proportion of current year's contributions) is 1.12%, the average annual growth rate of the lower bound of the 95% confidence interval (solid line at the bottom of the figure) is 0.73%, and the average annual growth rate of the upper limit is 1.80%, so its average annual growth rate of Indicator 1 will be in the interval [0.73%, 1.80%] with a probability of 95%.

Let the average annual change = (the value at the end of the forecast period – the value at the start of the forecast period) / the number of years in the forecast period. Let the annual average change rate = (the value at the end of the forecast period – the value of the year in which the payment gap occurs at the earliest) / the number of years between the end of the forecast period and the earliest year.

Then the average annual change in Indicator 2 (current year's balance of contributions and expenditures) is −1.41 trillion yuan, in the lower limit of the confidence interval is −12.05 trillion yuan, and in the upper limit of the confidence interval is −0.12 trillion yuan, so the average annual change of Indicator 2 is in the range [−12.05, −0.12] trillion yuan with a 95% probability. The annual average change rate in Indicator 3 (current year's payment gap as a proportion of current year's contributions) is 1.27%, in the lower limit of the confidence interval is 0.29%, and in the upper limit of the confidence interval is 3.89%, so the annual average change rate of Indicator 3 is in the range [0.29%, 3.89%] with a 95% probability.

Similarly, the average annual change in Indicator 4 (accumulated balance) is −31.70 trillion yuan, in the lower limit of the confidence interval is −133.39 trillion yuan, and in the upper limit of the confidence interval is −5.62 trillion yuan, so the average annual change of Indicator 4 is in the interval [−133.39, −5.62] trillion yuan with a 95% probability. Indicator 5 (fund ratio) indicates that the fund reserves will be exhausted in [2037, 2032] under pessimistic conditions (i.e., lower limit) and 2043 under optimistic conditions (i.e., upper limit). Thus, the fund reserves will be exhausted in [2032, 2043] with a 95% probability. The annual average change rate in Indicator 6 (accumulated payment gap as a proportion of current year's contributions) is 31.34%, and in the lower limit of the confidence and in the upper limit of the confidence interval, it is 6.72 and 215.63%, respectively, so the annual average change rate of Indicator 6 is in the interval [6.72%, 215.63%] with a probability of 95%. It can be seen that the fund reserve results obtained by stochastic simulation considering parameter uncertainty can cover various possibilities, including the results under the scenario of fixed-parameter value. While improving the forecast accuracy, more information on the changing trend in the fund reserves of basic pension insurance is provided.

**Figure 4:**
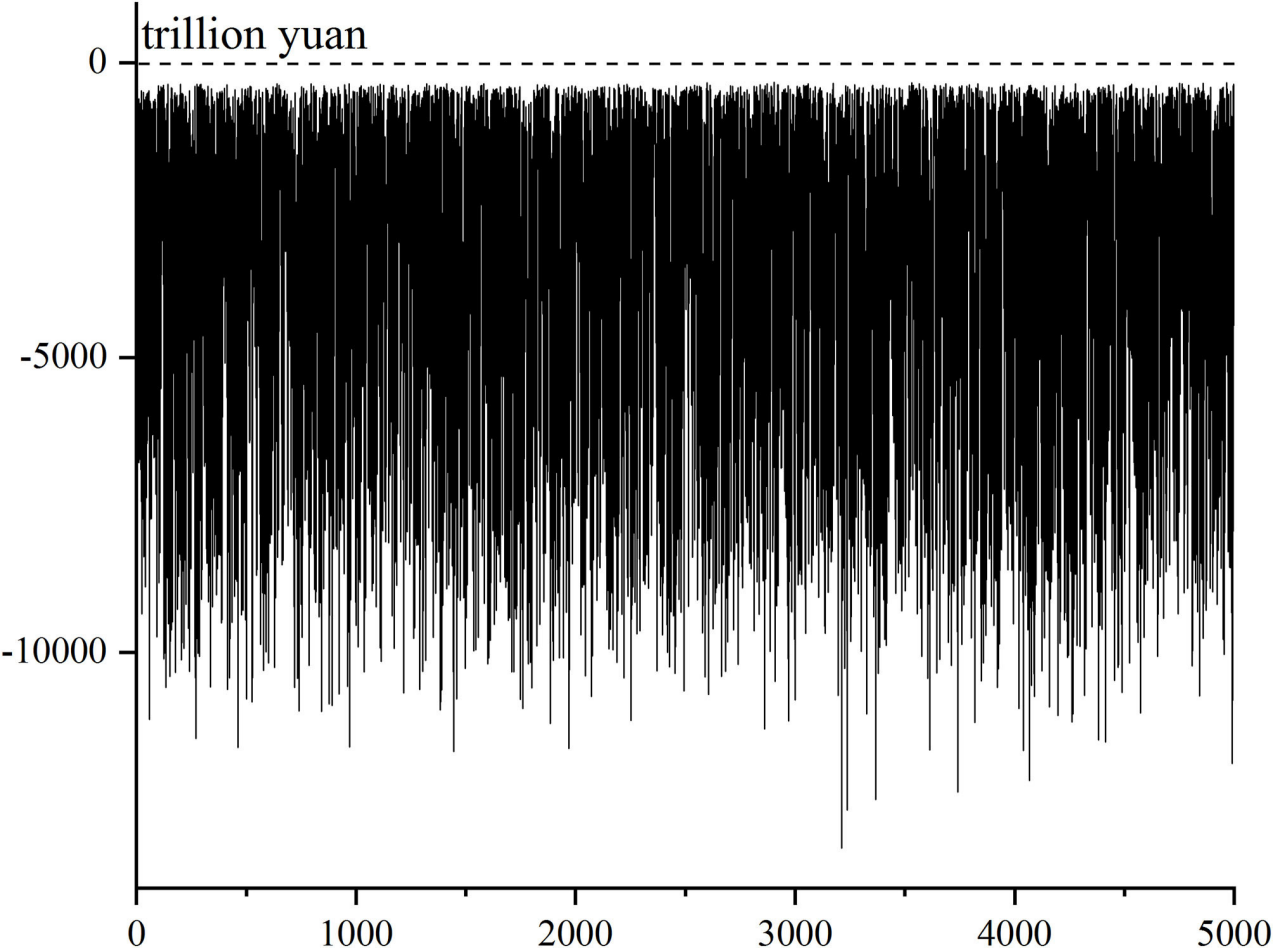
Distribution of the final fund reserve status of basic pension insurance.

The accumulated balance at the end of the forecast period measures the final state of the fund reserve of the basic pension insurance. Various possible states of the final fund reserve can be known from the accumulated balance in 2094 in the above 5,000 Monte–Carlo simulation results, and the results are shown in Figure 4.

From Figure 4, it is clear that in the relatively harsh environment without considering the transfer of state-owned capital, possible delaying retirement and financial subsidies in the future (i.e., isolating their beneficial effects) and the final fund reserve of basic pension insurance will be seriously insufficient in the prediction period.

## Further Analysis of Increasing Fund Reserves

Using stochastic technology to simulate various possibilities of the long-term fund reserves of basic pension insurance, it is found that the fund reserves will decline rapidly and will be exhausted in [2032, 2043] with a 95% probability, and the final fund reserve at the end of the forecast period will be seriously insufficient, indicating that the long-term fund reserves of basic pension insurance are extremely worrying. Thus, it is necessary to deeply grasp the relationship between these main parameters and the fund reserve status, so that the government can take measures to increase the fund reserves of the basic pension insurance.

### Impact Direction of Main Parameters on Fund Reserves

The calculation results of long-term fund reserves under the scenario of fixed parameter values are regarded as the benchmark case. Based on this situation, the sensitivity analysis of nine main parameters can be carried out to obtain the impact direction of these parameters on fund reserves. The sensitivity analysis of most existing literature is usually to change the value of a parameter once and analyze its influence compared with the benchmark case. However, a single change in parameter value can have different effects on fund reserves, which may destabilize the sensitivity analysis results. In order to avoid this kind of instability as much as possible, the influence of the continuous change of the parameter value is investigated here, and we obtained the relatively stable influence of the parameter.

The nine main parameters, such as mortality, unemployment rate, urbanization rate, etc., are continuously changed as follows:

the new values of the parameter = the value of the parameter under the benchmark case × (1+ τ%)

**Figure 5:**
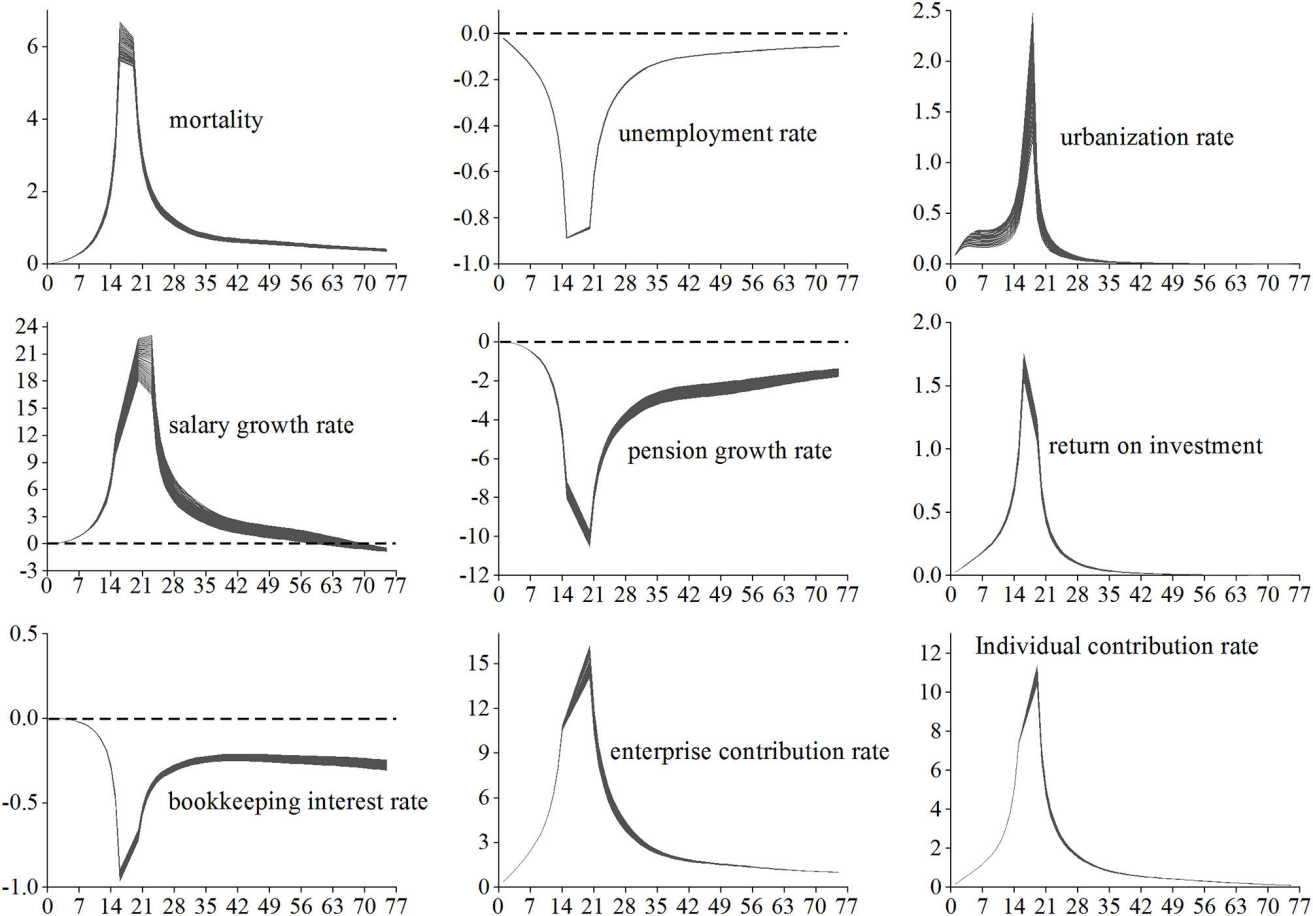
Elasticity of accumulated balance to each main parameter.

Where τ is [−25, −1] and [1, 25], which means that the parameter values gradually change from a uniform decrease of 25% to a uniform increase of 25%, that is, there are 50 sets of observations for each parameter. Using the change of Indicator 4 (accumulated balance) to examine the impact of parameter changes on the fund reserves, the elasticity of the accumulated balance to each main parameter is shown in Figure 5.

The influence directions of the nine main parameters can be divided into three categories. The first category includes the parameters that make the accumulated balance change in the same direction, such as mortality, urbanization rate, ROI, enterprise contribution rate, and individual contribution rate. The second category includes the parameters that reverse the change of accumulated balance, such as unemployment rate, pension growth rate, and bookkeeping interest rate. The third category includes the parameter that makes the accumulated balance change in the same direction first and then in the opposite direction, such as the average salary growth rate.

### The Influence Degree of Each Main Parameter on the Fund Reserves

The environment of basic pension insurance is complex and changeable, and the value of main parameters will be volatile. Based on the benchmark case, the influence degree of the random fluctuation of each main parameter on the fund reserves of the basic pension insurance is examined one by one. First, the the upper and lower limits of the 95% confidence interval of the fluctuation range of the accumulated balance in each year of the forecast period caused by the parameter fluctuation are obtained, then the upper limit is subtracted from the lower limit, and the mean value of the difference between these two limits is taken as the index *A* to measure the influence degree of the parameter on the fund reserve status.

For example, to calculate the influence degree of random fluctuations in mortality on fund reserves, first, the values of the remaining parameters are fixed, and only the mortality values that fluctuate, and 5,000 simulation operations of the fund reserve status are carried out. Second, the 5,000 simulation results of the accumulated balance from small to large are ranked, and the upper and lower limits of the 95% confidence interval of the accumulated balance in each year of the prediction period are obtained. Third, according to the meaning of index *A*, the influence degree of random fluctuation of mortality on fund reserves is calculated. Similar to the above calculation process, the influence degree of the fluctuation of each parameter on the fund reserve status is obtained. The influence degree of these main parameters is in the descending order: average salary growth rate, pension growth rate, unemployment rate, enterprise contribution rate, bookkeeping interest rate, mortality, individual contribution rate, ROI, and urbanization rate.

### Feature Search of Parameter Value Paths Under “Better” Fund Reserve Status

**Figure 6:**
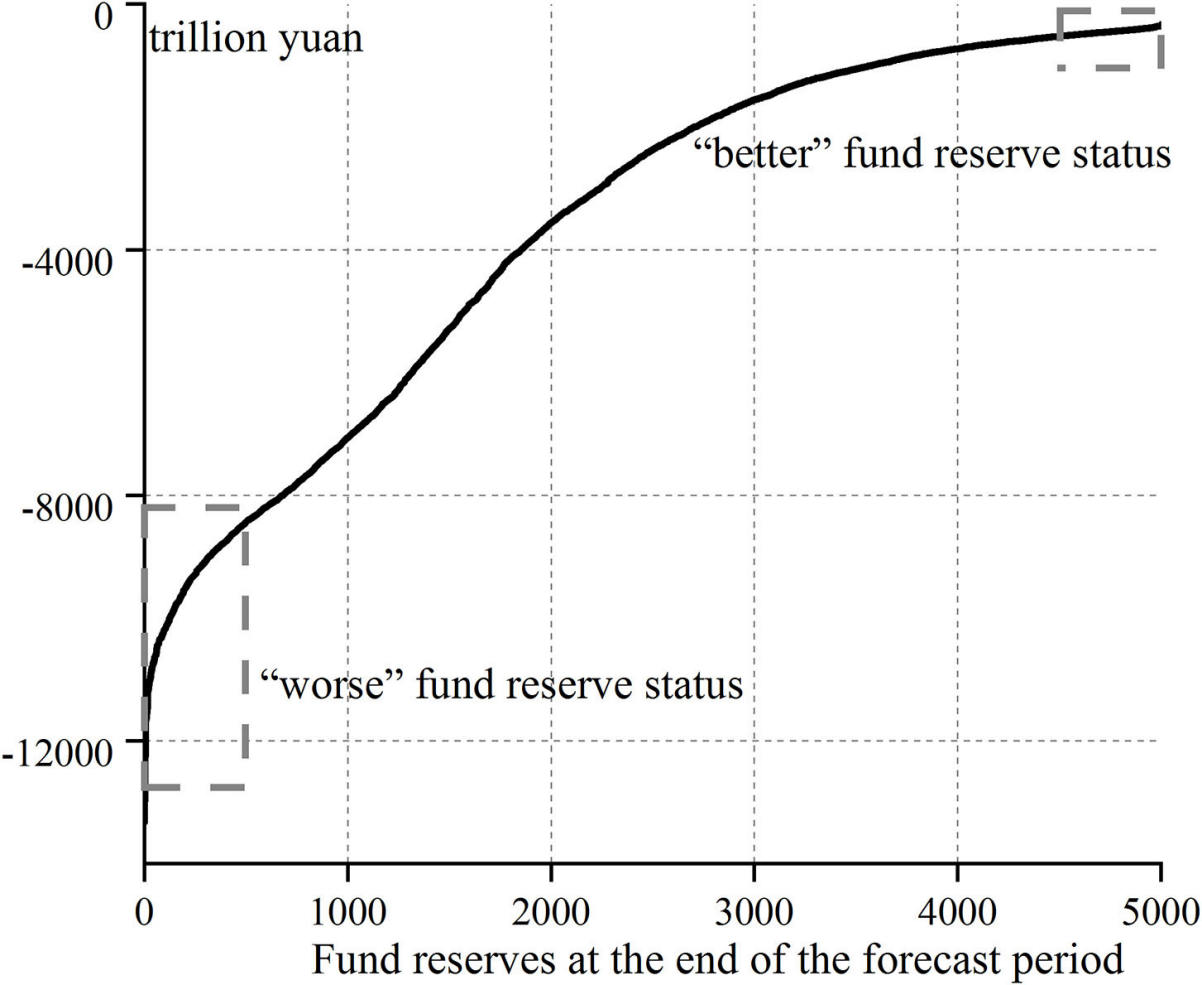
Schematic diagram of the meaning of “better”and “worse” fund reserve status.

The final fund reserve of the basic pension insurance in the forecast period in Figure 4 are sorted from small to large, and the results are shown in Figure 6, which can be divided into two different statuses. One is the fund reserve status above the 90% quantile (about 500 simulation results), with the minimum value of −524.63 trillion yuan, which is much larger than −3,187.41 trillion yuan in the benchmark case. Thus, these final fund reserves that are better than the benchmark case can be called “better” fund reserve status, which means that under the complex and changeable environment, these final fund reserves of basic pension insurance are lucky to occupy a more favorable position than the benchmark case. Similarly, the other is the fund reserve status below the 10% quantile, with the maximum value of −8,437.70 trillion yuan, which is far < −3,187.41 trillion yuan in the benchmark case, which is called the “worse” fund reserve status.

Then how does the basic pension insurance fortunately make its final fund reserve in a “better” position? That is, in a complex and changeable environment, how should the main parameters be valued so that the final fund reserve of the basic pension insurance is in a favorable position, and is there any obvious characteristic in the value path?

The concept of backtracking is introduced here. For any calculation result in random simulation, the value taking process of random parameters in each year of the prediction period can be traced back or recorded to form the parameter-value taking path corresponding to the result. We make a backtracking analysis on why the basic pension insurance is in the “better” fund reserve status and the “worse” fund reserve status, respectively and compared to find whether there are some characteristics in the parameter-value path.

**Figure 7:**
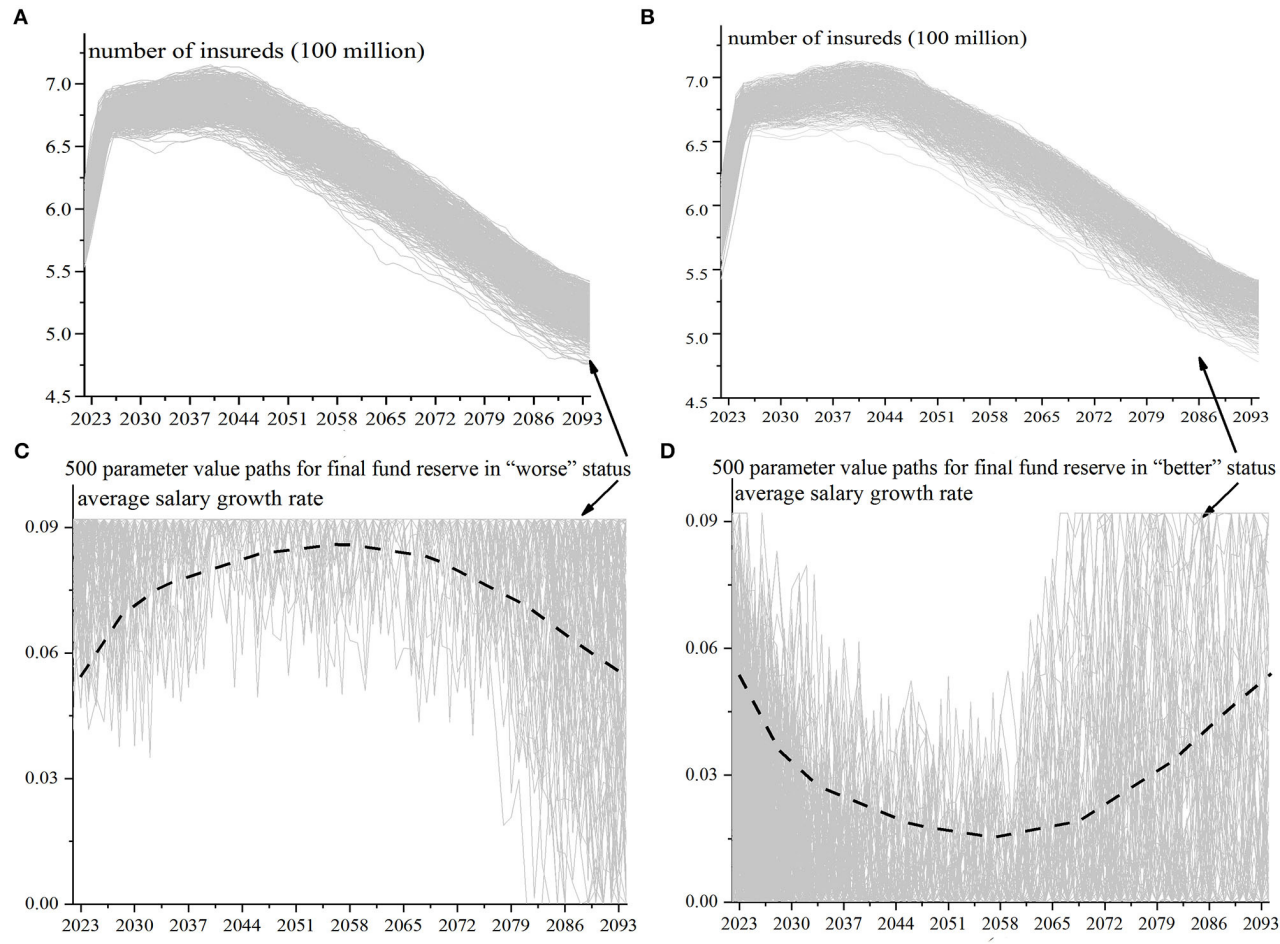
**(A–D)** Feature search of value paths for the number of insureds and average salary growth rate.

The fluctuation of mortality, urbanization rate, and unemployment rate can be comprehensively reflected in the fluctuation of number of insureds. For the 500 simulation results in which the final fund reserve is “better” and the 500 simulation results in which it is “worse” in Figure 6, the number of insureds, average salary growth rate, ROI, bookkeeping interest rate, enterprise contribution rate, and individual contribution rate are tracked back respectively. The value paths of the number of insureds and the average salary growth rate are shown in Figure 7. The Figures 7A,C are the parameter value paths when the final fund reserve status is in the “worse” position, and the Figures 7B,D are the parameter value paths in the “better” position.

There is no significant difference between the two value paths of the number of insureds, indicating that the random value paths of the number of insureds are almost the same if the final fund reserve status is in the “better” and “worse” position, and there is no value feature that causes the significant difference in the final reserve status. The two value paths of ROI, bookkeeping interest rate, enterprise contribution rate, and individual contribution rate are also similar. These are consistent with the number of insureds, and there is no significant difference. The average salary growth rate is different, and the two value paths are significantly different. As shown in Figure 7, if the value path of the average salary growth rate follows a trend of rising first and then falling, the final reserve status will be “worse”; on the contrary, if it follows a trend of first falling and then rising, the final reserve status will be “better”.

## Conclusions and Implications

To actively promote the consolidation progress of fund reserves of basic pension insurance and to contribute to the steady increase of social endowment wealth reserves, it is urgent to give priority to the stochastic evaluation of its future fund reserves. This study first analyzes the aging characteristics of the insureds of basic pension insurance and establishes the actuarial prediction model of contributions and expenditures for basic pension insurance based on the State Council Document 26 of 1997 and the State Council Document 38 of 2005 and considering the latest provisions of the *Comprehensive Scheme on Reducing Social Insurance Contribution Rates*. Then the nine main parameters are randomized, and various possibilities of the long-term fund reserve status of basic pension insurance are randomly simulated.

The study found that the aging of the insureds have two characteristics of “fierce coming and slow decline” and “long-term seriousness.” Among the six indicators to measure the fund reserve status of basic pension insurance, Indicator 1 (the current year's expenditures as a proportion of current year's contributions), Indicator 2 (current year's balance of contributions and expenditures), Indicator 3 (current year's payment gap as a proportion of current year's contributions), Indicator 4 (accumulated balance), Indicator 5 (fund ratio), and Indicator 6 (accumulated payment gap as a proportion of current year's contributions) are in the range of [0.73%, 1.80%], [−12.05, −0.12] trillion yuan, [0.29%, 3.89%], [−133.39, −5.62] trillion yuan, [2032, 2043] and [6.72%, 215.63%] with 95% probability respectively. What is more, under the harsh environment of not considering the transfer of state-owned capital, delaying retirement, and financial subsidies, the final fund reserve of the basic pension insurance will be seriously insufficient.

To improve the ability to consolidate the fund reserves, we analyze the influence of the main parameters on the fund reserves, their random fluctuations, and the influence of the parameter-value paths on the final status of the fund reserves. Sensitivity analysis identified three distinct categories of parameters affecting the direction of the fund reserves. In the first category, the parameters that make the accumulated balance change in the same direction include mortality, urbanization rate, ROI, enterprise contribution rate, and individual contribution rate. The second category is the parameters that make the accumulated balance change inversely, including unemployment rate, pension growth rate, and bookkeeping interest rate. The third category is the average salary growth rate for the parameters that make the accumulated balance change in the same direction and then in the opposite direction. Under the random fluctuation environment of future parameters, it is found that the influence degree of the fluctuation of main parameters on the fund reserves from large to small is the average salary growth rate, pension growth rate, unemployment rate, enterprise contribution rate, bookkeeping interest rate, mortality, individual contribution rate, ROI, and urbanization rate. The backtracking found that if the value path of the average salary growth rate showed a trend of rising first and then falling, the final reserve status would be “worse;” if it showed a trend of first falling and then rising, the final reserve status would be “better.”

According to the above research conclusions, in order to actively respond to the aging population and consolidate the fund reserves of basic pension insurance, the following inspirations can be obtained. First, to improve the long-term fund reserves of the basic pension insurance, the average salary growth rate should preferably show a trend of “falling first and then rising.” The above conclusions show that when the value path of the average salary growth rate in each year of the forecast period is “first fall and then rise,” the final fund reserve status will be “better.” The average salary growth rate belongs to the parameter of economic development, and the government has weak control over it. The overall future growth rate of China's economy may just show a trend of first falling and then rising. Although the current economic growth rate of China is gradually declining, the purpose of turning to high-quality economic development is to obtain a relatively high-speed and stable economic growth in the future, so it may show a downward trend and then an upward trend. Related studies show that the average salary growth rate and the economic growth rate fluctuate closely in tandem (8, 24). Thus, it is necessary to further strengthen the close link between the average salary growth rate and the economic growth rate and establish an adjustment mechanism in which the average salary growth rate fluctuates closely with the fluctuation of the economic growth rate.

Second, to steadily consolidate the fund reserves of basic pension insurance, the government should control the parameters of the basic pension insurance in a combined manner. The sensitivity analysis shows that the enterprise contribution rate and the individual contribution rate change in the same direction with the accumulated balance, while the pension growth rate and the bookkeeping interest rate change in the opposite direction with the accumulated balance. Thus, if the government wants to further reduce the payment burden of the basic pension insurance for enterprises without affecting the consolidation progress of basic pension insurance fund reserves, the government can try to take a combination of measures, such as moderately increasing individual contribution rate, reducing the pension growth rate, and bookkeeping interest rate.

Third, the ROI of pension is increased and the accumulated fund reserves of the basic pension insurance are consolidated. Sensitivity analysis shows that increasing the ROI will increase the accumulated balance in the same direction and enhance the pension payment ability to cope with the aging of the population. According to the “Basic Pension Insurance Fund Entrusted Operation Annual Report 2020”, the basic pension has been invested since 2016, and its annual average investment rate of return is 6.89%, but it fluctuates greatly. The lowest investment rate of return is 2.56% in 2018. Thus, it is urgent to actively study the investment strategies, investment fields, and investment supervisions of basic pension in order to stabilize its ROI.

Fourth, an actuarial reporting system of China's basic pension insurance should be established as soon as possible, and its fund reserve status should be regularly evaluated. Developed countries, such as the United Kingdom and the United States have already formed a mature financial evaluation system for pension funds. For example, the trustee of the “OASDI” fund in the United States submits the fund financial report to Congress every year, and the British government actuary reports the long-term financial report of the pension fund every 5 years. Under the background that the response to population aging has become a national strategy, relevant departments of China should regularly publish official actuarial reports on basic pension insurance or entrust relevant institutions to evaluate and monitor the fund reserves of basic pension insurance. By establishing the actuarial reporting system of China's basic pension insurance, we learn from the “OASDI” report of the United States and introduce stochastic technology to simulate the fund reserve situations in a complex and changeable environment as much as possible. At the same time, we try to introduce the concept of backtracking to find the possible path to improve the fund reserves of the basic pension insurance.

## Data Availability Statement

The original contributions presented in the study are included in the article/supplementary material, further inquiries can be directed to the corresponding author.

## Author Contributions

The author confirms being the sole contributor of this work and has approved it for publication.

## Funding

This research was supported by the Science and Technology Project of Jiangxi Provincial Department of Education (GJJ210542).

## Conflict of Interest

The author declares that the research was conducted in the absence of any commercial or financial relationships that could be construed as a potential conflict of interest.

## Publisher's Note

All claims expressed in this article are solely those of the authors and do not necessarily represent those of their affiliated organizations, or those of the publisher, the editors and the reviewers. Any product that may be evaluated in this article, or claim that may be made by its manufacturer, is not guaranteed or endorsed by the publisher.
